# Assessing the effects of variables and background selection on the capture of the tick climate niche

**DOI:** 10.1186/1476-072X-12-43

**Published:** 2013-09-26

**Authors:** Agustín Estrada-Peña, Adrián Estrada-Sánchez, David Estrada-Sánchez, José de la Fuente

**Affiliations:** 1 Department of Parasitology, Faculty of Veterinary Medicine, University of Zaragoza, Miguel Servet 177, 50013 Zaragoza, Spain; 2 Department of Geography, University of Zaragoza, Pedro Cerbuna, 2, 50006 Zaragoza, Spain; 3 SaBio, IREC, Ciudad Real, Spain; 4 Department of Veterinary Pathobiology, Center for Veterinary Health Sciences, Oklahoma State University, Stillwater, USA

**Keywords:** Ixodes ricinus, *Hyalomma marginatum*, Climate niche, MODIS, CliMond, WorldClim, Spatial autocorrelation, Collinearity

## Abstract

**Background:**

Modelling the environmental niche and spatial distribution of pathogen-transmitting arthropods involves various quality and methodological concerns related to using climate data to capture the environmental niche. This study tested the potential of MODIS remotely sensed and interpolated gridded covariates to estimate the climate niche of the medically important ticks *Ixodes ricinus* and *Hyalomma marginatum*. We also assessed model inflation resulting from spatial autocorrelation (SA) and collinearity (CO) of covariates used as time series of data (monthly values of variables), principal components analysis (PCA), and a discrete Fourier transformation. Performance of the models was measured using area under the curve (AUC), autocorrelation by Moran’s *I*, and collinearity by the variance inflation factor (VIF).

**Results:**

The covariate spatial resolution slightly affected the final AUC. Consistently, models for *H. marginatum* performed better than models for *I. ricinus*, likely because of a species-derived rather than covariate effect because the former occupies a more limited niche. Monthly series of interpolated climate always better captured the climate niche of the ticks, but the SA was around 2 times higher and the maximum VIF between covariates around 30 times higher in interpolated than in MODIS-derived covariates. Interpolated or remotely sensed monthly series of covariates always had higher SA and CO than their transformations by PCA or Fourier. Regarding the effects of background point selection on AUC, we found that selection based on a set of rules for the distance to the core distribution and the heterogeneity of the landscape influenced model outcomes. The best selection relied on a random selection of points as close as possible to the target organism area of distribution, but effects are variable according to the species modelled.

**Conclusion:**

Testing for effects of SA and CO is necessary before incorporating these covariates into algorithms building a climate envelope. Results support a higher SA and CO in an interpolated climate dataset than in remotely sensed covariates. Satellite-derived information has fewer drawbacks compared to interpolated climate for modelling tick relationships with environmental niche. Removal of SA and CO by a harmonic regression seems most promising because it retains both biological and statistical meaning.

## Background

Ticks are important vectors of pathogens to humans [1,2]. Most of the tick’s life cycle is spent in the environment, where ticks develop, moult, and quest actively for a host [3]. Temperature has a central role in the regulation of the tick life cycle, including the development of the moulting stages (or oviposited eggs) and the periods in which ticks quest for a host in the vegetation. During the winter, low temperatures prevent rapid development, so development progresses slowly until temperatures increase in spring. At northern latitudes, temperature is the main driving factor of the length of the tick life cycle by regulating the duration of developmental processes. The requirements of temperature for development are species-specific and commonly prevent the spread of ticks farther north, where total cumulative degrees in a year are too low to allow complete development. Mortality depends on water losses, which are regulated by the relative humidity and the air saturation deficit. During questing, ticks lose water that they normally regain by descending at intervals to the litter zone where they can reabsorb water vapour from the atmosphere [4,5]. When the ticks are hydrated, they ascend to the vegetation. The seasonal activity of ticks is characterised by several cycles of ascending and descending movements in the vegetation, regulated by temperature and water loss. Therefore, the energy reserves of the tick plus its abilities to retain water, together with air water content and temperature, are the factors regulating the questing and survival of ticks in the field.

Such tight dependence of ticks on climate traits makes them susceptible to meteorological changes, which in turn affects their periods of activity, development, and mortality and expansion into new zones or retreat from colonised areas [6,7]. Some of these shifts in distribution have been reported from field studies [8,9]. In other cases, associations between climate and prevalence rates of tick-transmitted pathogens have been proposed based on empirical grounds [10] or meta-analyses of published data [11]. However, the effect of the projected climatic trends over the rather complex life cycle of ticks and the dynamics of tick-transmitted pathogens are still poorly understood and subject to debate. Although a change in climate might play an important role in certain geographic regions, for much of Europe, non-climatic factors, such as host population dynamics, are becoming increasingly important in the recorded spread of the tick *Ixodes ricinus*[7]. Similar explanations have been hypothesized for the increase in prevalence rates of other tick-transmitted pathogens, in particular those carried by the tick *Hyalomma marginatum*[12].

Methods of species distribution modelling have been applied to arthropods of medical importance to understand the factors limiting their distribution [13-15]. These quantitative tools combine observations of species occurrence with environmental features [15] and are increasingly applied to produce coherent estimates of distribution patterns of mosquitoes [2], sandflies [16], and ticks [7,17]. The covariates of climate and vegetation with which these arthropods are associated can be used to gain information about the effects of future climate scenarios or even recent trends [18]. Because this information can be produced on a timely basis, with internally consistent data sources, it is a useful tool for resource managers, policy makers, and scientists interested in tracking recent changes across large administrative or environmental scales. These models are becoming increasingly popular in mapping the expected environmental variables that limit the physiological response of an arthropod vector [16].

Although some studies have emphasised the suitability of yearly averaged covariates involving temperature and rainfall, in the interpretation of the climate niche of the target arthropod [7,14], others have used sets of variables at monthly intervals or the orthogonal transformation of a time series of covariates, via principal component analysis (PCA) or Fourier transformation [19,20]. It has been explicitly indicated [21] that the set of covariates chosen to explain the abiotic habitat ought to have a clear biological meaning, describing adequately the biological and ecological constraints of the species in the spatial range to be modelled. Without this biological background, numerous variables can produce models with highly reliable matching distributions that are only statistically relevant. Although there is a tendency to consider that these potential distributions represent the probable geographical range, they must be regarded only as the characterization of the range of abiotic conditions (corresponding to non-living factors in the environment) under which the organism may survive [16]. These so-called “suitability maps” or “potential distribution species models” are interpretations of a similarity measure of the abiotic conditions at each pixel of the map with the conditions at the known range of the species. These maps are actually a projection into the spatial range of the inferences made on such a niche of the organism. Without a model aimed at describing every process of the life cycle of the target organism, it is necessary to carefully select the minimum set of covariates that adequately describe, without inflation, the variables driving the observed distribution.

A common problem in modelling the abiotic niche of arthropod vectors is the lack of assessment of the statistical issues derived from spatial autocorrelation (SA) and collinearity (CO) of the covariates. SA is the spatial co-variation of properties between records used for calibration of models [22] violating standard statistical techniques that assume independence among observations. SA thus arises from multiple points of “presence” for the organism to be modelled, not randomly distributed over the space [23]. Patterns of species distributions may be spatially autocorrelated because of population dynamics and historical factors, including closely clustered surveys that lead to the observed pattern of occurrence [24]. SA is thus a spatially related problem that leads to an overestimation of the sample size, inflating the statistical significance of the measured spatial relationships and increasing the likelihood of false positives (type I errors, [25]).

The problem of SA in the determination of the tick abiotic niche can be stated as follows. Consider a region of several square kilometres (i.e., representing a small fraction of its complete spatial range) where tick-transmitted pathogens are a concern and where active surveys for ticks are commonly carried out. The tick will be collected in such a range where the spatial variability of the climate covariates is low because it is a relatively small territory. These collections represent, however, a significant fraction of the complete distribution range of the tick, as reported, and contribute to populate the dataset of presences with closely located records having very similar abiotic “preferences”. This method biases the perceived niche of the tick because the tick has not been randomly collected in the context of the complete dataset. An additional problem is expected to arise when covariates are gridded interpolations of climate from recording stations. Consider the same territory for which only a few climate-recording stations exist. Even the best performing methods will interpolate a few points as a surface of data where large areas have almost the same values for the covariate. These surfaces are later used to know the niche at which ticks have been collected, which will result in a significant number of records in the tick presence dataset having similar values for the climate covariates and biasing our capture of its climate niche.

CO is a statistical phenomenon in which two or more covariates in a multiple regression model are highly correlated and presents a problem related to the internal structure of the covariates used to explain the distribution of the records. In our application, the typical situation involves the use of time series of covariates that are strongly correlated (e.g., the temperature in one month is expected to be very similar to the values of the following month). CO is thus a spatio-temporal problem originated in the structure of the covariates and not in the records used for calibration. A special situation exists when covariates are grid interpolations of climate point records. In this case, the problems are magnified because the interpolation algorithms use a set of discrete, irregularly spaced sites (the meteorological stations), and the temporal series of covariates will exhibit a high CO compared with the regularity and the continuity of remotely sensed measurements.

To take full advantage of the available resources, researchers need to know the extent to which different variables selected to drive the models may affect the final outcome. This study is aimed at identifying the optimal set of abiotic variables describing the environmental niche of the two prominent ticks *Ixodes ricinus* and *Hyalomma marginatum*. It was not a goal to evaluate the accuracy of different algorithms in producing different results or to produce ready-to-use maps. The overall aim rather was to gain a general knowledge of the main variables driving the distribution of these ticks and to identify some procedural gaps in the selection of the covariates because they are commonly targeted to sketch predictive maps applied to the improvement of human health. We explicitly sought to demonstrate that (i) no single method exists to produce the best map for ticks, (ii) covariates producing the best performing model have high colinearity and spatial autocorrelation, therefore rendering conclusions unreliable, and (iii) that the transformation of time series of covariates produce satisfactory results and remove most of the internal problems of covariates.

## Results

### Effects of data source

**Figure 1:**
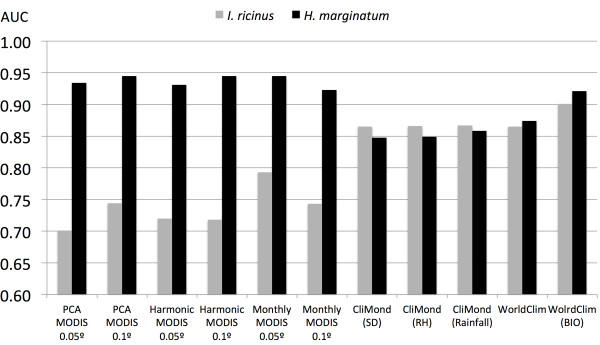
**AUC values of the models for either *Ixodes ricinus *or *Hyalomma marginatum*.** Models were built against a set of 10,000 random pseudo-absence records and trained with several sets of interpolated climate, using both temperature and saturation deficit (SD), temperature and relative humidity (RH), and temperature and rainfall in either the CliMond or WorldClim sets (rainfall) or the set of “Bio” variables derived from the WorldClim dataset (BIO). All the datasets were used at a spatial resolution of 0.1°.

Models for either *I. ricinus* or *H. marginatum* produced high AUC values, ranging from 0.7 to 0.9 (Figure 1). Worst results (lowest AUC) were consistently produced for *I. ricinus* using the set of remotely sensed covariates, in comparison with those for *H. marginatum* with the same sets of covariates. The resolution of the MODIS imagery had an influence in the results, with AUC values higher at lower resolution. Models based on monthly values of MODIS-derived data produced the highest AUC for the set of remotely sensed information. Models based on PCA and harmonic regression had almost similar AUC values for both resolutions of remotely sensed products. However, interpolated climate datasets produced high AUC values without important differences between species. Interpolated climate covariates also produced similar results for both species of ticks in terms of AUC (Figure 1), with slight differences among the different datasets used. The three sets of CliMond based on relative humidity, saturation deficit, and rainfall performed in similar terms. ROC curves for every model are included in Supplementary material.

### Background selection

**Figure 2:**
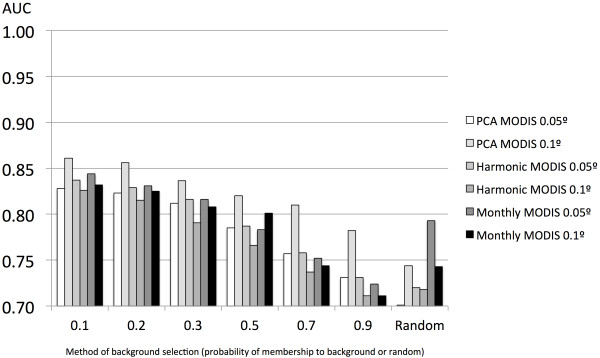
**AUC values of the models for *Ixodes ricinus*.** Pseudo-absence records were either a set of 10,000 random records, or selected at different membership values of the background or randomly distributed over the target territory. Models were trained with remotely sensed variables from MODIS imagery, at either 0.05° or 0.1° of spatial resolution. Covariates are the 12 monthly layers of surface temperatures and 12 other layers of NDVI (“Monthly”), the reduction of these monthly values by a principal components analysis (“PCA”) involving six covariates, and the coefficients of a harmonic regression (“Harmonic”) involving eight covariates.

**Figure 3:**
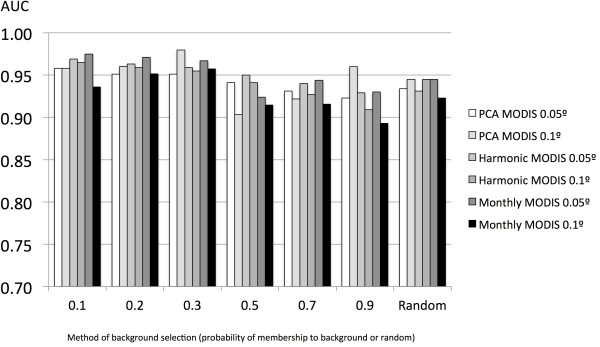
**AUC values of the models for *Hyalomma marginatum*.** Pseudo-absence records were either a set of 10,000 random records, or selected at at different membership values of the background or randomly distributed over the target territory. Models were trained with remotely sensed variables from MODIS imagery, at either 0.05° or 0.1° of spatial resolution. Covariates are the 12 monthly layers of surface temperatures and 12 other layers of NDVI (“Monthly”), the reduction of these monthly values by a principal components analysis (“PCA”) involving six covariates, and the coefficients of a harmonic regression (“Harmonic”) involving eight covariates.

The selection of the background affected the reliability of the models for *I. ricinus*, producing consistently higher AUC values if built against a background of points recorded as close as possible to its recorded distribution. Such a background represents the lowest range of the fuzzy membership function. Models built with a random background had an AUC about 12–15% lower than that with the optimal selection of the background points (Figure 2). This result was also observed for *H. marginatum*, but the relevance of the choice of the background points to the final AUC was less. The AUC of models for *H. marginatum* with a random selection of the background was only 2–4% lower than the optimal strategy of background choice (Figure 3).

### Spatial autocorrelation and collinearity

**Figure 4:**
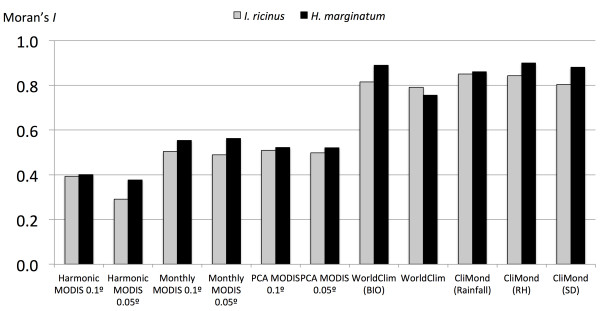
**Moran’s *I *values for the sets of records of either *Ixodes ricinus *or *Hyalomma marginatum*.**

Models developed for *I. ricinus* had lower values of SA, as measured by Moran’s *I*, than those for *H. marginatum*. This result was consistently obtained for each dataset of covariates (Figure 4). The lowest autocorrelation values were obtained when PCA or harmonic regression covariates of the MODIS datasets were used. The resolution influenced SA value, and covariates of smaller resolution had higher Moran’s *I* values when transformed by a harmonic regression. However, PCA transformations and monthly data had similar SA values. Higher values for Moran’s *I* were obtained for the monthly set of MODIS covariates. Interpolated climate datasets had consistently higher values of Moran’s *I* for each modelled species and every transformation (humidity, saturation deficit, or rainfall).

Values of VIF are included in Table 1 as the measure of CO of covariates. VIF was low for the MODIS monthly data transformed after PCA or harmonic regression, for both 0.05° and 0.1° of spatial resolution. Maximum values of VIF among any two of the MODIS-derived covariates was as low as 2.1 (theoretical upper limit being around 10). However, the set of monthly MODIS values displayed maximum VIF values of 177 and 189 for the resolutions 0.05° and 0.1°, respectively, meaning that at least two covariates of the series were highly correlated. Highest average VIF values were found for the monthly series of interpolated climate. The “Bio” series of layers derived from the WorldClim dataset displayed a mean VIF of 3.5, but several covariates were highly correlated between them, with maximum VIF values up to 196, some 20 times higher than the threshold indicative of a high CO.

**Table 1:** Mean, minimum, and maximum values of the variance inflation factor (VIF) as a measure of the collinearity observed in the different sets of variables

**Set**	**VIF: Average (Min/Max)**
Harmonic regression MODIS 0.1°	1.2 (1.0/3.3)
Harmonic regression MODIS 0.05°	1.1 (1.0/3.2)
Monthly variables MODIS 0.1°	8.2 (2.0/189.1)
Monthly variables MODIS 0.05°	8.3 (2.1/177.4)
PCA MODIS 0.1°	1.2 (1.1/2.2)
PCA MODIS 0.05°	1.1 (1.0/2.1)
WorldClim	12.2 (3.0/196.0)
WorldClim (Bio)	3.5 (1.0/125.4)
CliMond (Temperature + Rainfall)	12.1 (3.6/126.1)
CliMond (Temperature + RH)	12.4 (4.1/128.4)
CliMond (Temperature + SD)	13.5 (4.0/119.5)

The mean VIF averages such values for every pair-to-pair comparison between variables. Minimum and maximum values result from the absolute minimum and maximum comparisons: a high maximum VIF reflects high collinearity between, at least, two layers of covariates. A value of VIF = 10 is considered as the allowable maximum.

## Discussion

This study evaluated the use of different sets of informative variables (covariates) to estimate the climate suitability for two tick species with relevance for human health. We sought to provide a comparison of the suitability of several sets of climate covariates regarding the statistical issues of CO and SA [26-32]. We did not consider land features that might affect the abundance of hosts, which are necessary for the tick’s blood meal [33], or climatic extremes (cold spells, heat waves) that could limit tick survival. We thus focused on the estimation of systematic errors rather than on producing the best map of the expected distribution of these ticks’ ranges, as a preliminary step to computing the probable distribution of these species.

Although correlative models capture the distribution of a given organism in the n-dimensional environmental niche, the production of a “risk map” is only a projection from the environmental space into the geographical one. Both remotely sensed and interpolated climate datasets have advantages and disadvantages in the capture of such a niche. The first category has a high temporal resolution but a relatively short running period (typically since the years 1983 or 2000, according to the sensor), but satellite data must be corrected to remove clouds, ice, or artifacts [34]. Moreover, estimators of the saturation deficit are not easily available in the satellite products, although reports estimated the humidity component from standard NDVI imagery [35,36]. The interpolated climate datasets are easily available and free of contaminations like clouds or ice. These products are commonly available as the long-term average for the period 1960–1990 for the complete world, at different resolutions, and include estimations of water availability in the air, which are important for explaining tick activity and mortality rates [37]. A number of modellers have argued strongly for the use of predictors that are ecologically relevant to the target species [38-41]. It has been stated that the ‘use of automated solutions to predictor selection … should not be seen as a substitution for preselecting sound eco-physiological predictors based on deep knowledge of the bio-geographical and ecological theory’ [27], pp: 1681–1682]. In this context, the use of different variables describing the water content of the air (e.g., humidity or rainfall) has not affected the final outcome. However, these results have been obtained in the framework of a high CO of the covariates and are difficult to interpret.

It is important to mention that no single approach exists for the capture of climate niche of these species of ticks, at least with the two target species and the different sets of covariates used in this study. First, there is a clear effect derived from the species, which has been observed for remotely sensed information, but not for interpolated climate covariates. *I. ricinus* is a species colonizing a large area of the western Palearctic and thus reported under a large variety of environmental conditions [42]. A certain degree of adaptation of the tick populations to the regional climate conditions should therefore be expected, something that cannot be captured by the modelling algorithms because they work on the basis of the niche conservatism [43]. *H. marginatum* is a Mediterranean species, colonizing only the relatively warm and dry environments of the Mediterranean basin [17]. It is thus expected that adaptation to regional environmental conditions is lower than for *I. ricinus* because of the narrower region occupied in the environmental niche [17]. We ignore why this effect is not observed in the datasets of interpolated climate.

Studies simulating sets of pseudo-absences to train the models have tried to assess how the strategy of choice of background may influence the predictive abilities of models for organisms [43,44]. A large experiment [45] showed that a potential drawback of models generated with random pseudo-absences is that they might coincide with locations where the species actually occurs. This coincidence would strongly affect the calculation of the probability of presence in the model. Consequently, the models generated with random pseudo-absences are expected to have poorer fit [46]. The selection of the background is different for each target species and may depend upon the biology of each organism and the abiotic features of the territory to be modelled. For *I. ricinus*, a species that occupies a large portion of the available climate niche in the target region, the choice of a background near the recorded distribution of the tick (low membership probability) produced always better models, with a difference of about 12–14% of AUC values over the random background. For *H. marginatum*, which is restricted to a smaller volume of the available climatic conditions, such a choice of background affected model reliability in only 2–4% of AUC values. The selection of the background points from the n-dimensional distribution of the organism in its climate niche, and not from the spatial structure of its distribution, might be an interesting method to improve our understanding of the factors driving such distribution [47], a method that has not been addressed here.

Some sets of covariates tested for this study yielded high values of both SA and CO (as the Moran’s *I* and VIF). It was expected that the sets of interpolated climate resulted in higher SA values because these data are gridded interpolations of climate stations, with effects that are greater in regions where a low density of points is available, introducing uncertainties into the predictions. CO was also expected to be higher in datasets involving monthly covariates because each variable is correlated with others. Some studies [26,27,48] have recommended incorporating a term for SA into the analysis. However, this method has been criticized [29] because models that incorporate a SA term reflecting environmental rather than biological spatial structure could not be applied to other situations. Other approaches have involved the detection of autocorrelation among covariates before the modelling exercises, dropping the highly correlated covariates from the final modelling approach [49]. This method, however, might remove the most biologically important covariates because it is an automated solution that disregards the *a priori* ecological significance of covariates.

It seems, thus, that a single solution for the capture of the environmental niche of ticks is not straightforward. Remotely sensed information should be preferred to interpolated climate. However, in situations of a large number of pixels contaminated by water vapour, the use of interpolated climate should be considered, together with an explicit assessment of the CO of the residuals to check for inflation of the models. Monthly covariates of satellite-derived information had the highest values of VIF, but these values were clearly lower than the interpolated climate, including the set of “Bio” variables. Nevertheless, the models built upon the transformation of the original time series either by the coefficients of a harmonic regression or a PCA produced the best removal of SA and CO, as already reported [20]. The preferred method to infer the abiotic niche of these arthropods should be ideally based on a transformation of the original time series, extracting the raw temporal values in a series of uncorrelated covariates. It has been reported, however, that the PCA reduction of a time series lacks its intuitive meaning [21]. In contrast, a harmonic regression retains the terms about the amplitude and seasonality of each time component and is therefore easier to interpret. We advocate the use of the coefficients of a harmonic regression, which represent a summarized description of the climate niche of the organism while retaining the ability to explain seasonal trends with a few parameters. Moreover, the selection of variables derived from purely physical traits, like elevation, will contribute to inflate the results of the model by further CO with the raw climate features like the temperature. The inclusion of elevation is a common procedure in the building of correlative models, which cannot help in the interpretation of the niche of the organisms and will falsely inflate the predictive abilities of models [50].

## Conclusions

Several conclusions emerged from this study, and probably the most important is that no one method exists to elaborate maps of risk for arthropods of medical interest. Interpolated gridded climate covariates do not seem to be adequate tools for such a modelling exercise, even if they output high AUC values, because issues of SA and CO that may affect the reliability of the inference of the niche under some conditions. This also applies to time series of data (i.e. monthly intervals) that are obviously correlated, either for gridded or remotely sensed covariates. We might recommend the coefficients of a harmonic regression applied to the monthly series of remotely sensed information about LST and NDVI because they are uncorrelated covariates explaining the complete series. It is also important to investigate the effects of high-resolution features other than climate covariates (such as landscape composition or presence and abundance of suitable hosts) in the performance of the models. There is not a single method to select the background in presence-only models. There is an urgent need to adopt protocols to include real absence records of the ticks and to turn to statistical methods that can express the relationships of biological distributions with biologically meaningful climate covariates. It is concluded that procedures aimed to capture the distribution of arthropods with medical interest might be better focused on the inference about the climate niche, instead to simply project on the geographical distribution.

## Methods

### Explanatory variables and data preparation

The selection of the explanatory variables is a critical step in the inference and projection of the climate niche of an organism. In the case of ticks, additional complications arise because rain has little influence on the tick life cycle at large scales. The tendency is to correlate empirical observations on tick phenology with rainfall patterns; however, the factors affecting such phenology are temperature, relative humidity, and saturation deficit [4,51], and rainfall probably is adequate only at regional scales but unreliable for large patterns of variation. In the case of remote sensing, the Normalized Derived Vegetation Index (NDVI) is a variable that better captures the reported distribution of some species of ticks [37,52] because it is considered as a proxy for water availability.

We tested several sets of abiotic covariates at different resolutions and processed with different methodologies. Table 2 includes a list of the sets of raw abiotic variables and the further processing to obtain the variables driving the models. We used a set of MODIS satellite-derived, Land Surface Temperature (LST) and NDVI, at a spatial resolution of 0.05° (LST/NDVI A) or 0.1° (LST/NDVI B), obtained at a temporal resolution of either 8 (A) or 30 days (B), for the years 2000–2011. LST/NDVI A corresponds to the product MOD11C2, available at https://lpdaac.usgs.gov/products/modis_products_table (accessed May 2011) and contains the quality layers necessary to adequately assess the effects of clouds and aerosols on the image. Quality flags were addressed by removing pixels that were catalogued by MODIS as being obscured by clouds, water, or null/non-valid measurements. For every 8-day interval of the 2000–2011 period, we used only the pixels marked as “perfect”, “optimal”, and “valid but moderately affected by water vapour”, which yielded a set of monthly composites of either LST or NDVI. The monthly composites were obtained by the maximum pixel value of the 8-day products for such months.

**Table 2:** List of the datasets used in this study and the transformations carried out and applied to modelling purposes

**Set**	**Which variables**	**Resolution**	**Time**	**Covariates**
MODIS	LST and NDVI	0.05° and 0.1°	2000–2011	Monthly values: 24 variables
MODIS	LST and NDVI	0.05° and 0.1°	2000–2011	Principal components of the monthly datasets: 6 variables
MODIS	LST and NDVI	0.05° and 0.1°	2000–2011	Coefficients of harmonic regression of monthly values: 8 variables
WorldClim	Temperature and rainfall	10´	1960–1999	Monthly values: 24 variables
WorldClim	Temperature and rainfall	10´	1960–1999	Transformation of monthly values into “Bio variables”: 19 variables
CliMond	Temperature and relative humidity	10´	1960–1999	Monthly values: 24 variables
CliMond	Temperature and saturation deficit	10´	1960–1999	Monthly values: 24 variables

The basic set of data included the MODIS series of monthly values of Land Surface Temperature (LST) and Normalized Derived Vegetation Index (NDVI), as well as the interpolated gridded monthly climate data of temperature and rainfall or temperature, rainfall, and water saturation deficit.

LST/NDVI B is based on the MODIS product MOD11C2 and is available at http://neo.sci.gsfc.nasa.gov/Search.html (accessed December, 2011). This product has a smaller resolution than the original (0.1° instead of 0.05°) but is available as a ready-to-use set of monthly data, with the pixel contamination by water already removed by the MODIS team. The use of the data already processed by the MODIS processing team ensures its quality, significantly reduces the time for processing, and provides a clean set of data that can be directly used to feed the modelling chain of processes. We specifically tested it to check if its smaller resolution could affect the performance of the models.

These two products were used in three different ways: (1) averaged monthly values for the years 2001–2011 (12 variables for LST and 12 for NDVI); (2) the orthogonal transformations by PCA of the original series of data (the first 3 components for LST and the first 3 axes for NDVI, explaining 88.2% and 87.9% of the original variance, respectively); and (3) the coefficients of a harmonic regression of the series of data against time.

Harmonic regression is a mathematical technique used to decompose a complex signal into a series of individual sine and cosine waves, each characterized by a specific amplitude and phase angle [53]. In the process, a series of coefficients describe the cyclical variation of the series, including the seasonal behaviour. A variable number of components can be extracted, but only a few terms are in general necessary to describe annual, semi-annual, and smaller components of the seasonal variance. Such a procedure is similar to the transformation of a time series by a Fourier analysis [20], in which the harmonics of the series produce values for the maximum, phase, and period of the series. The harmonic regression was applied on the average of the 8- or 30-day MODIS images of LST or NDVI. The harmonic regression model used in this study was defined as follows:

$$Y=\beta_{0}+\mathrm{cT}+\sum_{i=1}^{n}A_{i}\sin\left(\frac{2\mathrm{\pi i}}{s}T\right)+\phi_{i}$$

where Y is the value of LST or NDVI, *B0* is the offset, c is the trend, Ai is the amplitude of the i^th^ oscillation, *ϕ* is the phase component of the i^th^ oscillation, s is the fundamental frequency, and T is the time-dependent variable. We performed both the PCA transformations and the harmonic regressions in R [54] and the package “raster” version 2.0-08 [55]. Four coefficients for LST and four for NDVI were used for model fitting because the addition of more terms did not significantly improve the fitting of the original series.

We also included monthly values of temperature and moisture obtained from two sets of interpolated gridded climate data, namely WorldClim [56] (available at http://www.worldclim.org) and CliMond [57] (http://www.climond.org). The former does not include data on the water content of the air but includes precipitation, and the latter includes both precipitation and relative humidity estimates. Although methods to interpolate the temperature are the same in both sets of data, the interpolation of the humidity features may differ. Therefore, we used both sets of data, namely temperature and precipitation, as available from WorldClim, and temperature and humidity, as available from CliMond. Details on the preparation of the datasets are available in references [56,57], respectively. In short, they include averaged monthly values for temperature and moisture (either precipitation or relative humidity) for the period 1960–1990, obtained from ground climate stations and interpolated thin-plate smoothing splines, using elevation, latitude, and longitude as independent variables.

We further processed the values of relative humidity as available in CliMond to obtain estimates of the saturation deficit because of the importance of such a feature in the life cycle processes of the ticks [51]. Monthly features of both sets were downloaded and used at a spatial resolution of 0.1° from their respective web sites (accessed February, 2012). We also used a set of variables derived from the main WorldClim dataset, which are called “Bioclimatic” variables and include information derived from the main monthly dataset. The bioclimatic variables represent annual trends (e.g., mean annual temperature, annual precipitation), seasonality (e.g., annual range in temperature and precipitation), and extreme or limiting environmental factors (e.g., temperature of the coldest and warmest months and precipitation of the wet and dry quarters). Interpolated climate covariates were not processed for further orthogonal transformations.

### Model building and comparison

For each species, models were developed with the sets of environmental covariates and a set of data point locations where the species has been observed in the western Palearctic. The ‘Maximum Entropy Approach’ using the MaxEnt computer program for modelling species geographic distributions (v.3.3.3k [58]) was employed to generate models for the species studied. The algorithm generates inferences from incomplete information, estimating a target probability distribution by finding the probability distribution of maximum entropy, subject to a set of constraints that represent the incomplete information about the reported distribution. This is a machine learning modelling method, which has recently gained attention for its favourable performance in comparison to other modelling methods [59]. We did not address a comparison of the reliability of the different modelling algorithms, and other methods using presence-only data are available [59].

**Figure 5:**
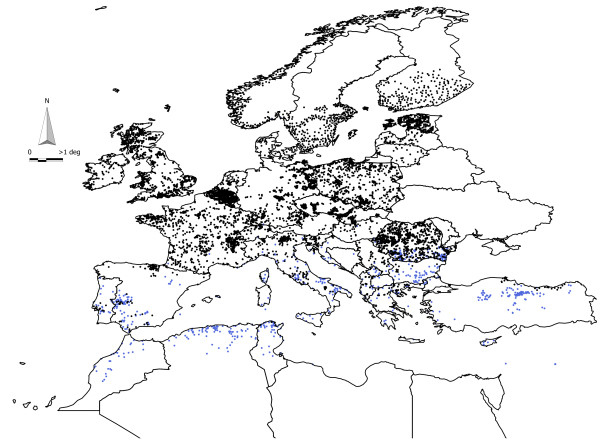
**The spatial distribution in the western Palearctic of the compiled records of the ticks *Ixodes ricinus *(black dots) and *Hyalomma marginatum *(blue dots) used to train the models.**

We used the reports of tick surveys as input data to train the models. These reports were previously compiled from different sources [17] and include 4,908 records of *I. ricinus* and 698 records of *H. marginatum* with a reliable geolocation. Figure 5 includes the spatial distribution of these data points in the target territory. More than 98% of the records were originally recorded for the period 1970–2010; however, some of the oldest records for *I. ricinus* were originally reported in the years 1910–1925. These records represent 1.5% of the total dataset and could affect the reliability of the captured niche because the periods of time of tick reporting and preparation of the climate dataset do not overlap [17]. This small fraction of old records was removed from the dataset before further analysis.

For each species, models were developed with every set of environmental covariates together with the set of data point locations where the species had been observed. We used quadratic and product terms to handle the non-linear response of ticks to climate covariates and to allow relationships among covariates to be included [49]. We explored a range of regularization parameters according to published recommendations [59] to choose a final regularization parameter (2 for both species to be modelled). Sampling bias was not addressed because of the inherent difficulties in its calculation on a historical dataset of records where no sampling effort was specifically included. Models were trained with 70% of records and evaluated against the remaining 30%. The modelled distributions were evaluated for predictive performance using the area under the receiver operating characteristic (ROC) curve to assess the agreement between the presence–absence records and the model predictions [60]. Model reliability was determined by calculating the area under the curve (AUC) such that a curve that maximizes sensitivity for low values of the false-positive fraction is considered a good model. The AUC represents a reliable important metric for evaluating diagnostic procedures, providing a single measure of model reliability, independent of any particular choice of threshold value [60]. Since its first proposal as an appropriate method to estimate the accuracy of species distribution models, reports have described its use in this field of research [61-63]. However, other studies have criticised its indiscriminate application [45,64]. We calculated and compared AUC using the R package pROC [65]. Additional file 1: Figure S1 and Additional file 2: Figure S2 include the ROC curves for the models. Additional file 3: Figure S3 and Additional file 4: Figure S4 include maps of the climate similarity for either *Ixodes ricinus* or *Hyalomma marginatum* in the target territory.

### Selection of pseudo-absences

The lack of a set of negative (absence) records—a usual bias in surveillance information—makes it necessary to compute pseudo-absences, which are randomly selected over the background of the target territory. The modelling software selects a random set of background points; however, the wide range of environmental conditions under which pseudo-absences can be located might severely alter/bias the outputs of the calculations [66,67]. We elaborated on a method to select a background, comparing the AUC values obtained by the default random selection of absences provided by MaxEnt software against those built employing a customized, ad-hoc approach that select negative records from the background on the basis of criteria with ecological meaning. To do so, a grid with cells at a resolution of 0.01 degrees was created for the study area, and the probability for each pixel to be selected as background was calculated on a cell-by-cell basis. The rationale is that each cell of the grid has a probability to be a “background site”, which depends on both the distance to the nearest record of the target organism and the terrain ruggedness. The terrain ruggedness is defined as the difference in elevation between adjacent cells in a digital elevation grid covering the area of study. This is a standard definition (Topographic ruggedness index [68]) aimed to produce an evaluation of how variable the terrain in the cell is. A digital elevation model at a resolution of 1 km (obtained from http://www.worldclim.org, accessed December 2011) was used to compute the landscape heterogeneity, using a script for ArcGIS Desktop (ESRI, Redlands, CA, USA) available at http://arcscripts.esri.com (Topographic ruggedness index, accessed January, 2012).

**Figure 6:**
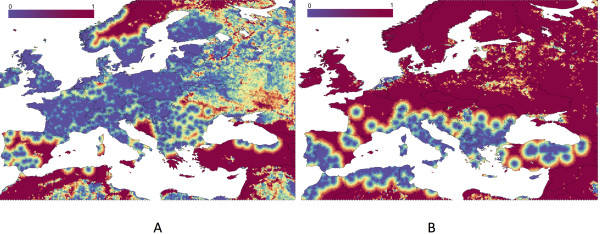
**The membership function used to select the background to train the models for *Ixodes ricinus *(A) and *Hyalomma marginatum *(B).** The tone from blue to red shows the degree of membership to the background (pseudo-absence) records and is based on the landscape heterogeneity and distance to known records of the target tick.

A fuzzy membership operator was applied to the distance-to-presences and terrain ruggedness variables to derive for each cell the probability to be selected as background. Mean and maximum distances within each set of records were the values of 0% and 100% of membership for “distance”. Mean and 75th percentile values of ruggedness were the values of 0% and 100% of membership for “ruggedness”. If the target cell is “far” from a cell where the organism has been recorded, the tick is probably absent in the candidate cell, and that cell has a high probability of being a background site. The probability of being background decreases as terrain ruggedness increases, meaning that the target cell may contain populations of the tick not yet surveyed. Figure 6 provides a visual explanation of the values of background membership, ranging from 0 to 1, for either *I. ricinus* (2A) or *H. marginatum* (2B) over the target territory. We produced models with background points based on different degrees of membership to the background, from 0.1 to 0.9, and each combination of remotely sensed covariates. Models were also built using a set of 10,000 randomly selected background points and compared against the models built with background points selected as described before. The influence of background selection on AUC values was done only for the set of remotely sensed covariates and their transformations.

### Assessing spatial autocorrelation and collinearity

We assessed the SA and CO of the sets of explanatory variables used in this study. The effect of SA on linear regression significance values has been tested using artificially generated variables with known spatial structures [69-71]. A common procedure to cancel the effect of the spatial structure of species occurrences is to incorporate a term for SA into the analysis [26,27,48], usually a measure of contagion that encompasses the effect of spatial neighbourhood in the statistical test. However, spatial patterns observed in the residuals could just as well result from failure to include an important autocorrelated predictor in the model [28] as much as from a real biological process. As a result, models that incorporate a SA term reflecting environmental rather than biological spatial structure will hardly be applicable to other situations [29].

On the other hand, it has been reported that CO does not affect MaxEnt performance (i.e., the predicted distribution range of the target species) but can hinder model interpretation (i.e., obstruct the decision about the covariates driving the distribution of the target species) [30]. The focus here is not the reliability of MaxEnt as affected by CO but to demonstrate that such a spatial effect exists in the sets of covariates most commonly used to map the distribution of arthropods that affect human health. In most cases, it is important to know how each variable influences the presence of the modelled species and, subsequently, which variables have the greatest influence on the model and in what manner these variables influence species occurrence [31]. Caution must be used when assessing this importance because a strong CO can influence results by implying greater importance for one of two or more highly correlated variables.

We used the Moran’s *I* for the model residuals to assess the SA of the covariates, separately for each set of covariates and respective transformations, the different spatial resolutions, and the two sets of tick records used to train the models [71]. We computed Moran’s *I* with the module “Autocorr” in Idrisi for Windows (V14) on the first lag only so that the algorithm scans through the whole image of model residuals and looks at each cell and its immediate neighbours. Moran’s *I* ranges between −1 and +1, where +1 means absolute and 0 no spatial autocorrelation. A negative index could indicate some kind of regular pattern. We assessed CO of the covariates with the variance inflation factor (VIF), which is a measure of correlation between pairs of variables [72]. Values of VIF > 10 denote a potentially problematic CO within the set of covariates, indicating that these covariates should be carefully evaluated in model development [32]. VIF was calculated for every combination of covariates and their orthogonal transformations and resolutions. Results are presented as the mean, minimum, and maximum values of VIF found in every pair combination of covariates among each set.

## Competing interests

The authors declare that they have no competing interests.

## Authors’ contributions

AEP and ECDC’s scientific staff (see acknowledgments) conceived the protocols. AEP developed models. AEP and AES built up the GIS system and processed the raw data. AES and DES processed satellite images and did most of the modelling. AEP and JF discussed the results and wrote the paper. All authors read and approved the final version of the paper.

## Supplementary Material

Additional file 1: Figure S1 ROC curves for the models built with different sets of remotely sensed variables and selecting the background according to the membership values of a fuzzy logic set of rules. The comparison with the ROC curves as obtained by a random selection of the background is included. A, B, C, D: *Hyalomma marginatum*; E, F, G, H: *Ixodes ricinus*. The sets of remotely sensed variables are as follows: Harmonic regression from MODIS data at 0.05° (A, E) and 0.1° (B, F); monthly values of the MODIS series (C, G) and a transformation by a principal components analysis over the monthly series of values of MODIS at 0.1° (D, H). Click here for file

Additional file 2: Figure S2 ROC curves for the models built with different sets of interpolated gridded climate. A: *Hyalomma marginatum*. B: *Ixodes ricinus*. Click here for file

Additional file 3: Figure S3 Maps of climate similarity in the target territory (from 0 to 100) for *Ixodes ricinus* produced by four different sets of variables. A: WolrdClim using 12 months of averaged temperatures and 12 months of averaged precipitation; B: MODIS monthly values, using 12 months of LST and 12 months of NDVI. C: Transformation of MODIS monthly values by a harmonic regression (Fourier transformation) using the first coefficients of LST and the first 5 coefficients of NDVI. D: PCA transformation (3 axes) of MODIS monthly values. Click here for file

Additional file 4: Figure S4 Maps of climate similarity in the target territory (from 0 to 100) for *Hyalomma marginatum* produced by four different sets of variables. A: WolrdClim using 12 months of averaged temperatures and 12 months of averaged precipitation; B: MODIS monthly values, using 12 months of LST and 12 months of NDVI. C: Transformation of MODIS monthly values by a harmonic regression (Fourier transformation) using the first coefficients of LST and the first 5 coefficients of NDVI. D: PCA transformation (3 axes) of MODIS monthly values. Click here for file
