# Supplementary figures and images for: Assessing the effects of variables and background selection on the capture of the tick climate niche
Source: Int J Health Geogr. 2013 Sep 26;12:43. doi: 10.1186/1476-072X-12-43 (PMC3849650; doi:10.1186/1476-072X-12-43)

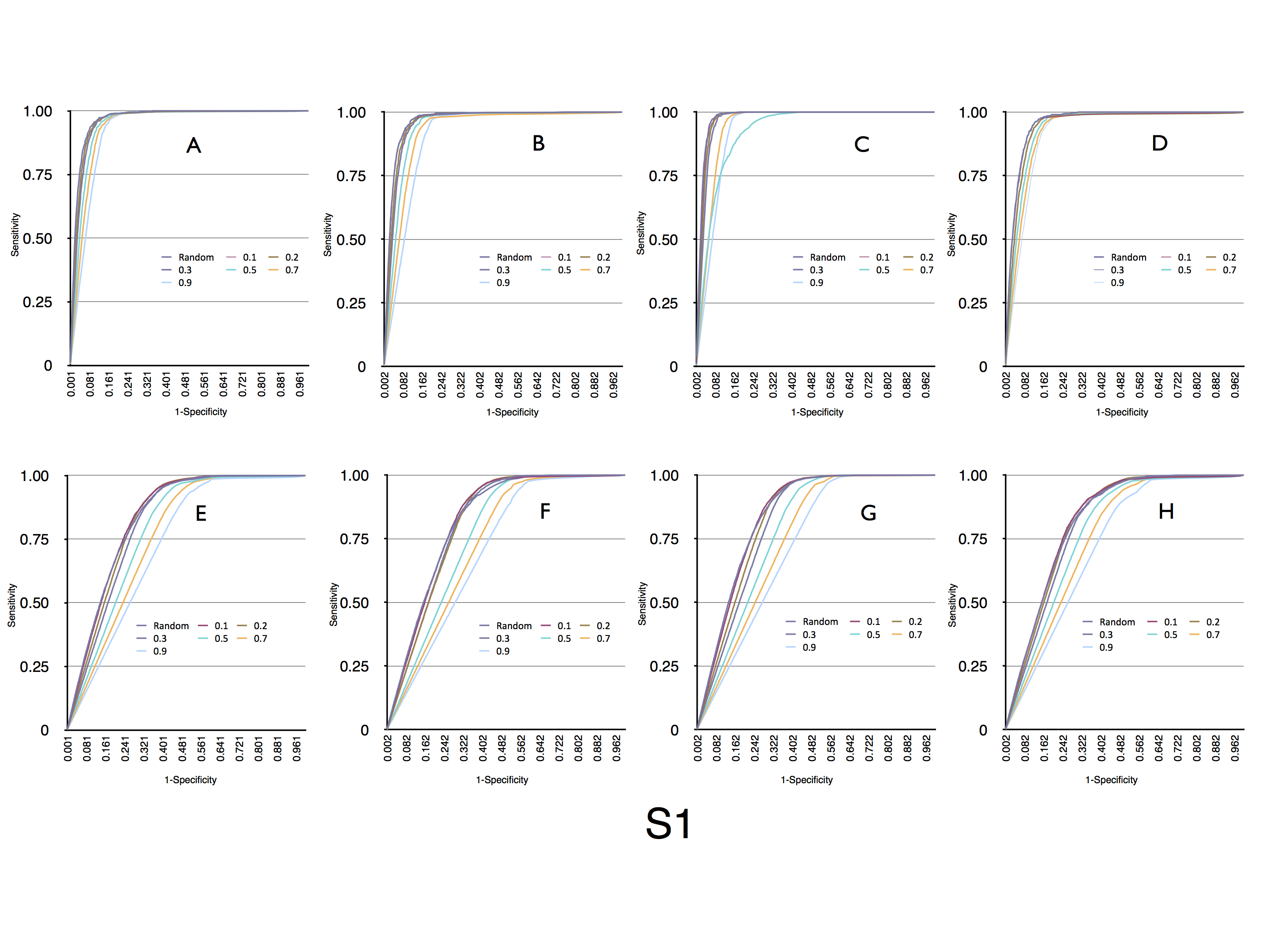

Supplement: Additional file 1: Figure S1 — ROC curves for the models built with different sets of remotely sensed variables and selecting the background according to the membership values of a fuzzy logic set of rules. The comparison with the ROC curves as obtained by a random selection of the background is included. A, B, C, D: Hyalomma marginatum; E, F, G, H: Ixodes ricinus. The sets of remotely sensed variables are as follows: Harmonic regression from MODIS data at 0.05° (A, E) and 0.1° (B, F); monthly values of the MODIS series (C, G) and a transformation by a principal components analysis over the monthly series of values of MODIS at 0.1° (D, H). [file 1476-072X-12-43-S1.png]

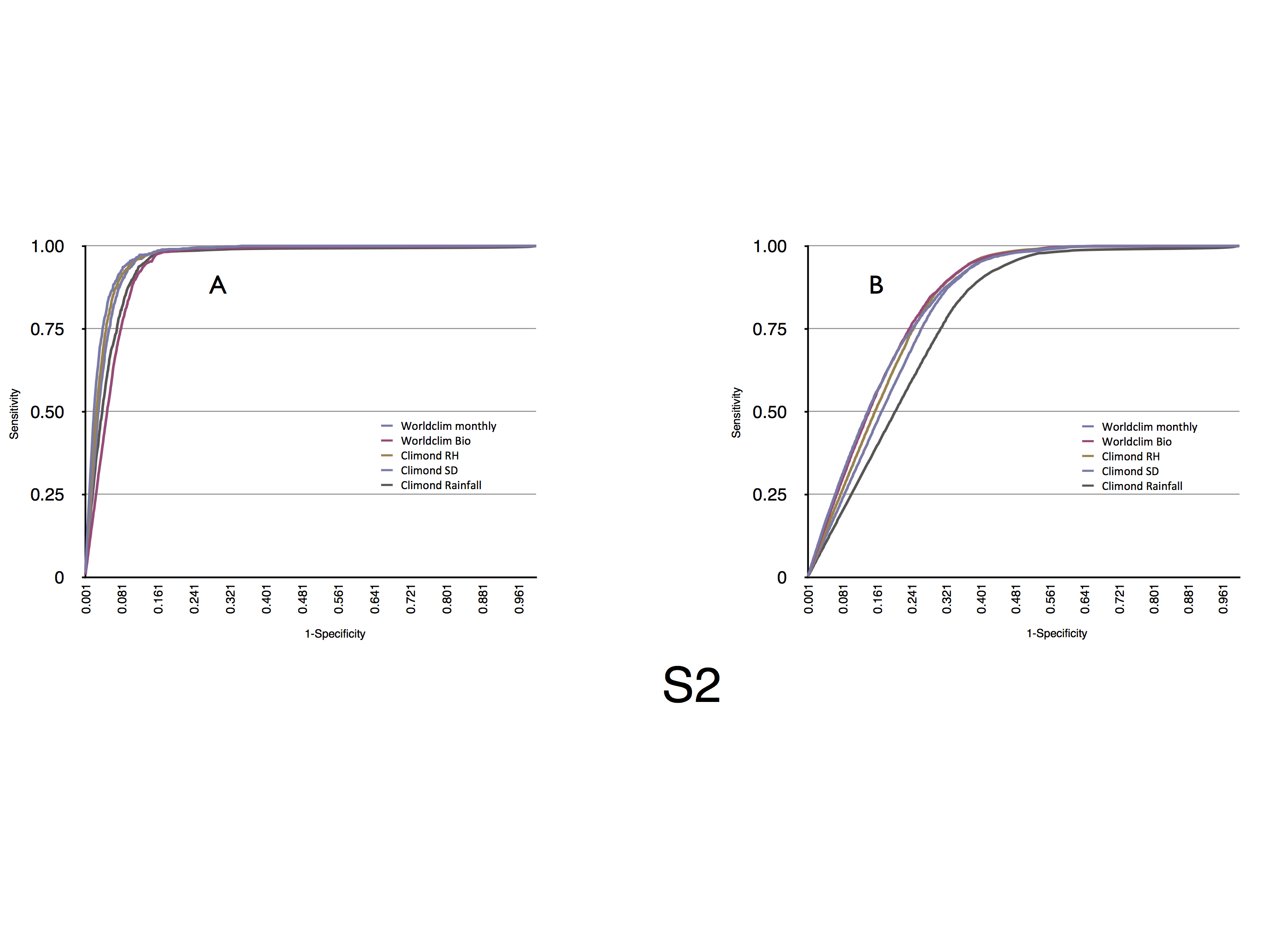

Supplement: Additional file 2: Figure S2 — ROC curves for the models built with different sets of interpolated gridded climate. A: Hyalomma marginatum. B: Ixodes ricinus. [file 1476-072X-12-43-S2.png]

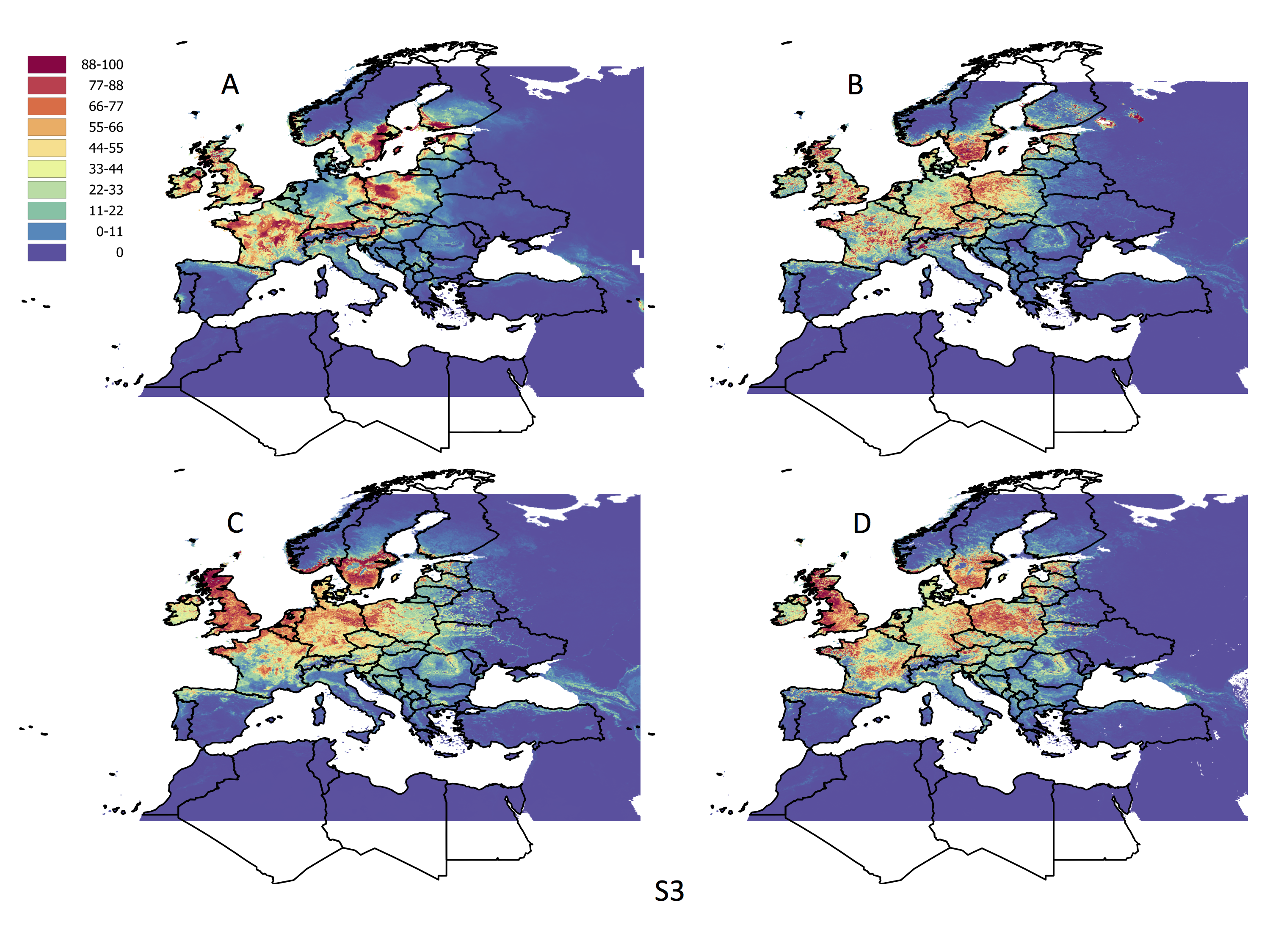

Supplement: Additional file 3: Figure S3 — Maps of climate similarity in the target territory (from 0 to 100) for Ixodes ricinus produced by four different sets of variables. A: WolrdClim using 12 months of averaged temperatures and 12 months of averaged precipitation; B: MODIS monthly values, using 12 months of LST and 12 months of NDVI. C: Transformation of MODIS monthly values by a harmonic regression (Fourier transformation) using the first coefficients of LST and the first 5 coefficients of NDVI. D: PCA transformation (3 axes) of MODIS monthly values. [file 1476-072X-12-43-S3.png]

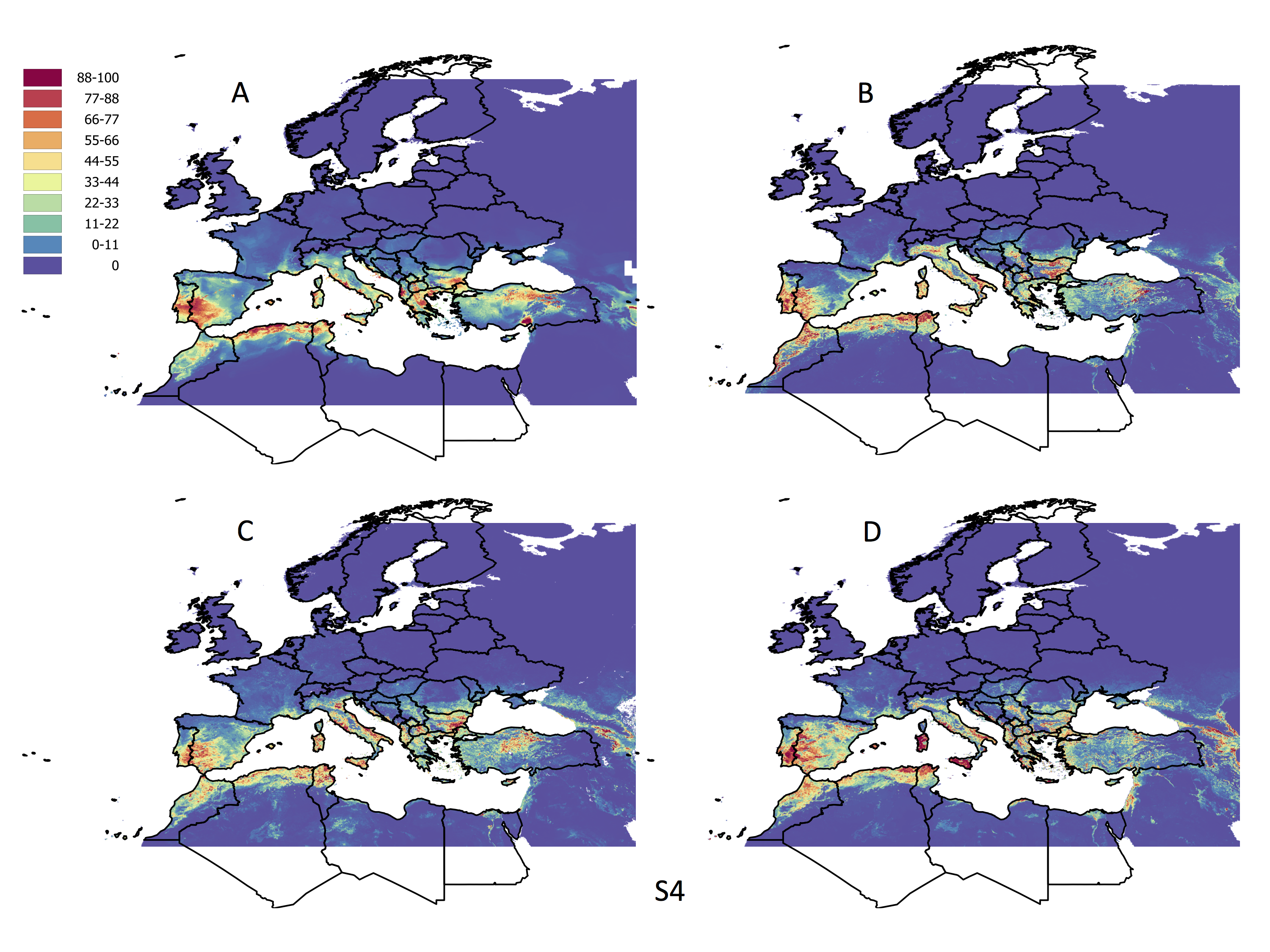

Supplement: Additional file 4: Figure S4 — Maps of climate similarity in the target territory (from 0 to 100) for Hyalomma marginatum produced by four different sets of variables. A: WolrdClim using 12 months of averaged temperatures and 12 months of averaged precipitation; B: MODIS monthly values, using 12 months of LST and 12 months of NDVI. C: Transformation of MODIS monthly values by a harmonic regression (Fourier transformation) using the first coefficients of LST and the first 5 coefficients of NDVI. D: PCA transformation (3 axes) of MODIS monthly values. [file 1476-072X-12-43-S4.png]
